# A new technique of laparoscopic fixation of the uterus to the anterior abdominal wall with the use of overfascial mesh in the treatment of pelvic organ prolapse

**DOI:** 10.1007/s00192-020-04287-4

**Published:** 2020-09-28

**Authors:** Jakub Śliwa, Anna Kryza-Ottou, Aleksandra Zimmer-Stelmach, Mariusz Zimmer

**Affiliations:** 2nd Department of Gynecology and Obstetrics, Wroclaw Medical University, Borowska 213, 50-556 Wrocław, Poland

**Keywords:** Pelvic organ prolapse, Uterine fixation, Hysteropexy, Uterine prolapse, Mesh, Surgical video

## Abstract

**Introduction and hypothesis:**

Pelvic organ prolapse is one of the most common pathological conditions in postmenopausal women. There is still a lack of fully effective and safe surgical techniques, especially in the advanced stages of apical defects. The purpose of the video is to present a new technique of laparoscopic treatment in women with an advanced stage of genital prolapse, stage III and IV according to the POP-Q scale. The technique involves uterine fixation for the anterior abdominal wall using overfascial mesh.

**Methods:**

We used a live-action surgical demonstration to describe laparoscopic fixation of the uterus to the anterior abdominal wall with the use of overfascial mesh.

**Results:**

This video provides a step-by-step approach to laparoscopic fixation of the uterus to the anterior abdominal wall with the use of overfascial mesh. The video can be used to educate and train those performing female pelvic reconstructive surgery.

**Conclusions:**

Based on our experience, this technique of laparoscopic suspension of the uterus to the anterior abdominal wall with the use of overfascial mesh is an effective, safe, and easy procedure for the treatment of advanced stages of pelvic organ prolapse.

**Electronic supplementary material:**

The online version of this article (10.1007/s00192-020-04287-4) contains supplementary material. This video is also available to watch on http://link.springer.com/. Please search for this article by the article title or DOI number, and on the article page click on ‘Supplementary Material’

## Introduction

Pelvic organ prolapse (POP) affects nearly half of women over the age of 50 and is one of the most common female pathological conditions [1]. Among the most frequent symptoms are the following: a sensation of a mass bulging into the vagina, a sensation of something coming or falling out of the vagina, urinary incontinence, fecal incontinence, having to push up on the perineum or digitate the vagina in order to urinate or defecate, and discomfort during sexual intercourse [2].

Anamnestic factors such as the method of delivery, newborn birth weight, and BMI most strongly affect the occurrence of prolapse in the future [3, 4].

Despite many known techniques of surgical treatment of genital prolapse, there is still no method that would be fully effective, safe, and technically simple with a short learning curve. Vaginal and abdominal access procedures are performed using native tissue (Kelly colpoplasty, McCall culdoplasty) or synthetic materials (sacrocolpopexy, pectopexy) [5].

In recent years, attention has been paid to the risk of complications associated with the use of synthetic materials—meshes in pelvic reconstructive surgery. Commonly reported adverse events included mesh exposure (0–39%), urinary retention (0–80%), and sexual dysfunction (0–48%). [6–8].

On 16 April 2019, the FDA ordered all manufacturers of surgical mesh intended for the transvaginal repair of anterior compartment prolapse (cystocele) to stop selling and distributing their products immediately owing to the lack of evidence concerning the safety and effectiveness of these devices. The safety of the meshes used in transabdominal POP surgery and urinary stress incontinence are still being analyzed [9]. In addition, a trend has been observed in recent years in line with long-term follow-up and patient preferences—patients opt for uterine-sparing POP treatment [10]. The number of vaginal hysterectomies decreased and the number of uterus-preserving operations increased between 2010 and 2016 [11].

The purpose of this video is to demonstrate laparoscopic fixation of the uterus to the anterior abdominal wall with the use of overfascial mesh in a woman with POP at stage III on the POP-Q scale. In the surgical technique presented here, the mesh was used to strengthen the weakened fascia of the anterior abdominal wall, but it was implanted outside the peritoneal cavity—over the fascia of the abdominal muscles. Placement of the mesh in this way ensures safety and efficiency, but at the same time excludes the risk of mesh adhesion and erosion in the peritoneal cavity.

Using live footage and step-by-step instructions, this video serves as an educational tool for female pelvic reconstructive surgeons of all levels to understand and learn this new surgical procedure.

## Materials and methods

Approval by the Commission of Bioethics at Wroclaw Medical University, Wrocław, Poland was obtained for this procedure.

This video uses live surgical footage to demonstrate a new technique of laparoscopic fixation of the uterus to the abdominal wall with the use of two non-resorbable sutures and overfascial mesh. The mesh was implanted over the fascia in order to strengthen it and to provide support for the sutures. Placement of the mesh in this way ensures safety and efficiency, but also excludes the risk of mesh adhesion and erosion in the peritoneal cavity. A suspension multifilament non-absorbable suture was used, but a monofilament suture such as polypropylene can be also considered. The operation is associated with sparing of the uterus. If the uterus is larger than average or there are some small to medium-sized uterine fibroids, this procedure can also be performed.

The following steps are key to successfully performing the procedure and are highlighted in the video:

- Step 1: insertion of a manipulator through the cervix into the uterine cavity for better mobility of the uterus.
- Step 2: typical laparoscopic access to the peritoneal cavity is obtained. One optical trocar and two lateral trocars are used. Bilateral adnexectomy is performed because of the presence of bilateral para-ovarian cysts.
- Step 3: a small transverse skin incision is made two fingerbreadths above the pubic bone to the level of the fascia. Subcutaneous tissue blunt dissection is performed to the level of the fascia.
- Step 4: first transperitoneal—uterine suture. The first non-absorbable thread on a straight needle is pierced through the fascia, the straight muscles, and the peritoneum into the peritoneal cavity. The needle (laparoscopic view) is then punctured through the uterus at the height of the isthmus from front to back and in the reverse direction from back to front. It is then punctured through the peritoneum, straight muscles, and fascia on the outside. The assumed suture is U-shaped. Manipulator movement can be seen to be helpful for operators when applying sutures using a straight needle to the uterus.
  The second transperitoneal–uterine suture is applied using a straight needle. It is applied in the same way as the first in order to strengthen the suspension of the uterus. When applying the sutures in the peritoneal cavity, the frontal bladder borders should be observed for safety reasons, so that the bladder wall is not damaged by a needle.
- Step 5: fixation of the overfascial mesh. A 4- × 2-cm polypropylene mesh is placed directly on the previously dissected fascia, under the subcutaneous tissue. Uterine sutures are placed over the mesh and tied together. When the sutures are tied up, the uterus is pulled up to the abdominal wall with its front surface in the anterior direction. The sutures of the subcutaneous tissue and skin are then tied. The patient did not experience any severe lower abdominal pain at the mesh and suspension site. There is a possible risk of enterocele formation after pulling the pelvic organs anteriorly in some patients, but this can be successfully eliminated by performing a posterior vaginal wall colpoplasty.

After the procedure, long-term urinary tract symptoms were not noticed, except for a clear improvement of urination in women with advanced stages of genital prolapse and urinary retention before surgery.

This video is a preliminary presentation of a new surgical technique and a publication presenting short- and long-term results of this type of surgery is being collected.

## Conclusion

Based on our experience, this technique of laparoscopic anterior abdominal wall fixation of the uterus with the use of overfascial mesh is an effective, safe, and easy procedure for the treatment of advanced stages of POP.

Using live footage and step-by-step instructions, the video serves as an educational tool for female pelvic reconstructive surgeons who, owing to restrictions on the use of meshes, are searching for a new surgical procedure.

## Electronic supplementary material

ESM 1 (MP4 336,687 kb)
